# Buoyancy effects on the radiative magneto Micropolar nanofluid flow with double stratification, activation energy and binary chemical reaction

**DOI:** 10.1038/s41598-017-13140-6

**Published:** 2017-10-10

**Authors:** M. Ramzan, Naeem Ullah, Jae Dong Chung, Dianchen Lu, Umer Farooq

**Affiliations:** 1 Department of Computer Science, Bahria University, Islamabad Campus, Islamabad, 44000 Pakistan; 2 Department of Mathematics, Quaid-i-Azam University, Islamabad, Pakistan; 3 Department of Mechanical Engineering, Sejong University, Seoul, 143-747 Korea; 4 Faculty of Science, Jiangsu University, Zhenjiang, Jiangsu China; 5 Department of Mathematics, COMSATS Institute of Information Technology, Park road, Tarlai Kalan, Islamabad, 45550 Pakistan

## Abstract

A mathematical model has been developed to examine the magneto hydrodynamic micropolar nanofluid flow with buoyancy effects. Flow analysis is carried out in the presence of nonlinear thermal radiation and dual stratification. The impact of binary chemical reaction with Arrhenius activation energy is also considered. Apposite transformations are engaged to transform nonlinear partial differential equations to differential equations with high nonlinearity. Resulting nonlinear system of differential equations is solved by differential solver method in Maple software which uses Runge-Kutta fourth and fifth order technique (RK45). To authenticate the obtained results, a comparison with the preceding article is also made. The evaluations are executed graphically for numerous prominent parameters versus velocity, micro rotation component, temperature, and concentration distributions. Tabulated numerical calculations of Nusselt and Sherwood numbers with respective well-argued discussions are also presented. Our findings illustrate that the angular velocity component declines for opposing buoyancy forces and enhances for aiding buoyancy forces by changing the micropolar parameter. It is also found that concentration profile increases for higher values of chemical reaction parameter, whereas it diminishes for growing values of solutal stratification parameter.

## Introduction

In mixtures, species with varied concentration are responsible for mass transfer processes. In such cases, migration of species is observed to an area of low concentration from the region of high concentration. Processes involving mass transfer phenomenon are diffusion of nutrients in tissues, absorption, thermal insulation and food processing. Mass transfer process with chemical reaction has been an area of interest for scientists and researchers for last many years because of many important applications like nuclear reactor cooling, geothermal reservoirs and thermal oil recovery. To name a few, Chamkha *et al*.^1^ studied the natural convection boundary layer flow problem along a cone in the presence of radiation effects and chemical reaction. Mallikarjuna *et al*.^2^ analyzed the chemical reaction effects on the flow and heat transfer of viscous nanofluid along a vertical cone in a variable porous medium. Ramzan and Bilal^3^ discussed the flow behavior of an elastico-viscous nanofluid affected by chemical reaction and magnetic field in three dimensions over a stretching sheet.

The term activation energy was initially presented by Svante Arrhenius in 1889^4^. It is characterized as the base measure of energy required for reactants to change into products. All molecules have energy, it can be in the form of kinetic or potential energy. The energy of molecules can be utilized to stretch, twist and ultimately to break bonds which leads to a chemical reaction. When molecules move gradually with minimum kinetic energy or slam into improper orientations, they do not react and simply bounce off each other. However, a reaction occurs when the momentum of molecules is sufficiently quick, to such an extent that kinetic energy of impact is more than the base energy barrier. Thus, the minimum energy requirement for a chemical reaction to take place is called activation energy. We can write the modified Arrhenius equation as (see Tencer *et al*.^5^).

1 $${K}_{r}=B{(\frac{T}{{T}_{\infty }})}^{n}\exp [\frac{-{E}_{a}}{{K}_{1}T}],$$

in which *K*_*r*_ is the rate constant and *B* is the pre-exponential factor or simply a constant, *E*_*a*_ is the activation energy, $({K}_{1}=8.61\,\ast \,\frac{{10}^{-5}ev}{K})$ is the Boltzmann constant and *n* is the fitted rate constant that lies in the range −1 to 1. The idea of activation energy is usually pertinent in the area related to geothermal or oil repository engineering, in the hydrodynamics and oil emulsion. In the recent years, the combined effect of chemical reaction and activation energy have been investigated by several researchers. A simple model with binary chemical reaction in the boundary layer flow over a plate was initiated by Bestman in^6^. Makinde *et al*.^7^ worked out the unsteady boundary layer flow of nanofluid over a porous plate under the action of radiation and chemical reaction. Maleque in^8^ and^9^ described the mixed convection boundary layer flow of nanofluid under the action of binary chemical reaction and activation energy. In another study, Awad *et al*.^10^ presented the effects of chemical reaction and Arrhenius activation energy on unsteady rotating nanofluid flow over a stretching sheet. He adopted special relaxation method (SRM) to interpret the problem. Later on, Shafique *et al*.^11^ investigated the flow of Maxwell nanofluid with combined effects of chemical reaction and activation energy in a rotating frame. Mustafa *et al*.^12^ reported numerical investigations of visco-elastic fluid flow influenced by a magnetic field and chemical reaction activated by energy. Another attempt in this area was made by Abbas *et al*.^13^. They scrutinized numerically the flow of Casson nanofluid past a shrinking/stretching surface at stagnation region in the presence of thermal radiation, binary chemical reaction and activation energy effects.

Scientists determined that the heat transfer rate can be triggered by making use of nanoparticles in liquids. Pioneering work in this regard was done by Choi^14^ who introduced the concept of liquids with nanoparticles. Buongiorno^15^ established that the thermophoretic diffusion and Brownian motion of nanoparticles are the significant mechanisms for the abnormal convective heat transfer improvement. Analysis of nanofluid flows have an incredible reputation in the field of research in the modern era because of its scope in power generation, as a coolant in vehicles, in refining the proficiency of refrigerant, and in certain biomedical applications, as it is utilized in the analysis of tumors and in other areas. Numerous studies in this field were made by many researchers, amongst them Chamkha and Aly^16^ numerically discussed convection flow of fluid with nanoparticles along a vertical plate with magnetic field effects, suction or injection, and heat generation or absorption. Ferdows *et al*.^17^ established the mixed convection boundary layer flow of nanofluid through a porous medium in the presence of magnetic field over an exponentially stretching surface. Makinde^18^ carried out the MHD flow of heat transfer of nanofluid near a stagnation point past a stretching/shrinking sheet. Effects of magnetic field and thermal radiation in the flow of Jeffrey nanofluid with thermal and solutal stratification was discussed by Ramzan *et al*.^19^. In another study, Haq *et al*.^20^ analyzed the convective flow of micropolar nanofluid along a vertically stretching surface in the presence of buoyancy forces and thermal radiation. Noor *et al*.^21^ scrutinized the effects of mixed convection and slip in the stagnation flow of micropolar nanofluid along a vertically stretching surface. Effects of mixed convection and thermal radiation in the Oldroyd-B fluid flow along a stretched surface are reported by Hayat *et al*.^22^. Recently Othman *et al*.^23^ presented numerically calculated convective boundary layer flow of nanofluid along a vertical stretched surface near a stagnation point. Besthapu *et al*.^24^ studied mixed convective nanofluid flow due to a vertical exponentially stretched surface in the presence of magnetic effect, thermal radiation and viscous dissipation. Ellahi *et al*.^25^ worked out the mixed convection flow of nanofluid with different size nanoparticles suspended in HFE-7100 over a wedge with entropy generation effect. Some more noteworthy explorations highlighting significance of nanofluids in varied flows may be found at^26–37^.

From the aforementioned studies, it is gathered that present exploration is unique and no such study has been carried out in the literature to date as far as combination of boundary layer flow of micropolar nanofluid along the vertical stretched surface in the presence of buoyancy forces, chemical reaction and Arrhenius activation energy effects. Heat and mass transfer processes are illustrated in the presence of Lorentz forces and double stratification. Numerical solution of the problem is obtained using a numeric differential solver method in Maple which utilize Runge-Kutta fourth and fifth order technique. To validate the accuracy of our study, a comparison is made with the previous article by Mustafa *et al*.^38^ and all results are found in good agreement.

## Theory and Flow Field Analysis

**Figure 1:**
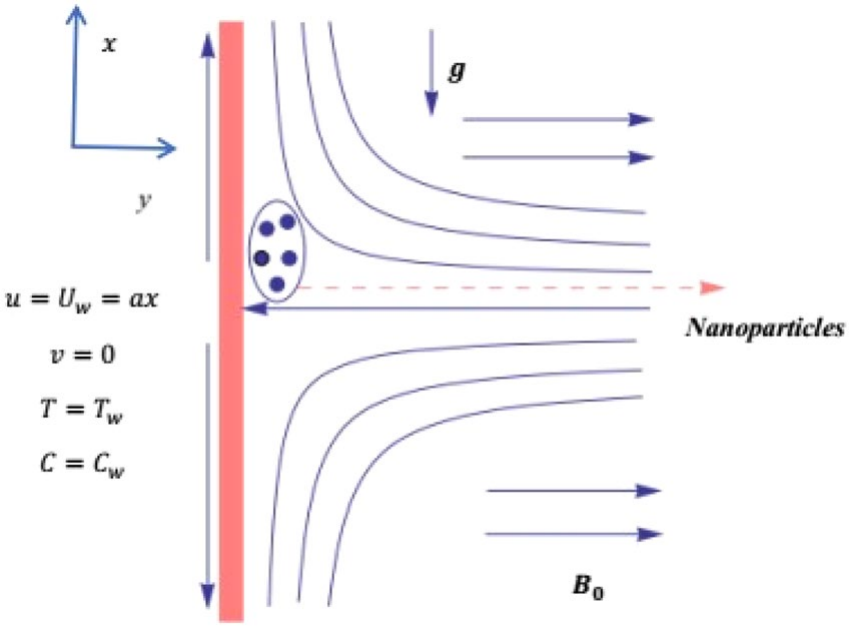
Diagram of flow problem.

Consider MHD flow of the micropolar nanofluid along a vertical stretched surface. The problem is characterized under the action of non-linear thermal radiation along with thermal and solutal stratifications. The combine effect of chemical reaction and activation energy is taken into account. The coordinate system is taken as *x*-axis along the surface and *y*-axis is normal to it as displayed in Fig. 1. A uniform magnetic field of strength *B*_0_ is applied normal to the flow. The surface stretches linearly in the vertical direction with velocity *U*_*w*_ = *ax* where *a* > 0 represents the stretching rate constant. The temperature *T*_*w*_ and concentration flux *C*_*w*_ are at the wall of the surface, while the ambient temperature and concentration are ${T}_{\infty }$ and ${C}_{\infty }$.

In the view of these assumptions the problem is governed by the following equations (see ref.^20,38^).

2 $$\frac{\partial u}{\partial x}+\frac{\partial v}{\partial y}=0,$$

3 $${\rho }_{f}(u\frac{\partial u}{\partial x}+v\frac{\partial v}{\partial y})=(\mu +k)\frac{{\partial }^{2}u}{\partial {y}^{2}}+k\frac{\partial N}{\partial y}-\sigma {{B}_{0}}^{2}u+(1-{C}_{\infty }){\rho }_{f}\beta (T-{T}_{\infty })g-({\rho }_{p}-{\rho }_{f})g(C-{C}_{\infty }),$$

4 $${\rho }_{f}j(u\frac{\partial N}{\partial x}+v\frac{\partial N}{\partial y})=\gamma \frac{{\partial }^{2}N}{\partial {y}^{2}}-k(2N+\frac{\partial u}{\partial y}),$$

5 $$u\frac{\partial T}{\partial x}+v\frac{\partial T}{\partial y}={\alpha }_{f}\frac{{\partial }^{2}T}{\partial {y}^{2}}+\tau [{D}_{B}(\frac{\partial C}{\partial y}\frac{\partial T}{\partial y})+\frac{{D}_{T}}{{T}_{\infty }}{(\frac{\partial T}{\partial y})}^{2}]-\frac{1}{{(\rho {c}_{p})}_{f}}\frac{\partial {q}_{r}}{\partial y},$$

6 $$u\frac{\partial C}{\partial x}+v\frac{\partial C}{\partial y}={D}_{B}\frac{{\partial }^{2}C}{\partial {y}^{2}}+\frac{{D}_{T}}{{T}_{\infty }}\frac{{\partial }^{2}T}{\partial {y}^{2}}-{{K}_{r}}^{2}(C-{C}_{\infty }){(\frac{T}{{T}_{\infty }})}^{n}\exp [-\frac{{E}_{a}}{{K}_{1}T}],$$

where *u* and *v*are the velocity components along *x*-and *y*-direction, *μ*, *k*, *σ*, *β*, *g*, *ρ*_*f*_ and *ρ*_*p*_ are the dynamic viscosity, vortex viscosity, electrical conductivity, thermal expansion coefficient, acceleration due to gravity, density of fluid and nanoparticles respectively. *N* is angular velocity component, *j* and *γ* are micro inertia density and spin gradient viscosity. *T* is the temperature *α*_*f*_, *D*_*B*_, *D*_*T*_, $\tau =\frac{{({\rho }_{f}c)}_{p}}{{({\rho }_{f}c)}_{f}}$ and *q*_*r*_ are the thermal diffusivity of the base fluid, Brownian diffusion coefficient, thermophoretic diffusion coefficient, the ratio of the effective heat capacity of the nanoparticle material to heat capacity of the fluid and radiation heat flux. *C* is the concentration field,$\,{C}_{\infty }$ the ambient particle concentration. Whereas *K*_*r*_, *E*_*a*,_ *K*_1_ and *n* are chemical reaction constant (rate constant), activation energy parameter, Boltzmann constant $({K}_{1}=8.61\,\ast \,\frac{{10}^{-5}ev}{K})$ and fitted rate constant respectively.

The boundary conditions are as follows:

$$u={U}_{w}=ax,\,v=0,N=-m\frac{\partial u}{\partial y},\,T={T}_{w}={T}_{0}+bx,\,C={C}_{w}={C}_{0}+cx\,\mathrm{at}\,y=0,$$

7 $$u\to 0,\,N\to 0,\,T\to {T}_{\infty }={T}_{0}+dx,\,C\to {C}_{\infty }={C}_{0}+ex\,\mathrm{as}\,y\to \infty .$$

In the above expressions *T*_0_, *C*_0_ and *a*, *b*, *c*, *d* are the reference temperature, reference concentration and dimensionless constants. *m* is the micro-gyration parameter, it specifies variations in concentration like, when *m* = 0 it indicates strong concentration and it means that the microelements close to the surface are unable to rotate; for *m* = 0.5 the anti-symmetric part of stress tensor vanishes and denotes weak concentration; while *m* = 1.0 modeled the turbulent boundary layer flows.

Making use of Rosseland approximation of radiation, the net radiation heat flux is simplified as

8 $${q}_{r}=-\frac{4{\sigma }^{\ast }}{3{k}^{\ast }}\frac{\partial {T}^{4}}{\partial y}=-\frac{16{\sigma }^{\ast }}{3{k}^{\ast }}\frac{{T}^{3}\partial T}{\partial y}$$

where *σ* ^*^ and *k* ^*^ are the Stefan-Boltmann constant and mean absorption coefficient respectively. Using Eq. (8) the temperature equation takes the form

9 $$u\frac{\partial T}{\partial x}+v\frac{\partial T}{\partial y}=\frac{\partial }{\partial y}[({\alpha }_{f}+\frac{16{\sigma }^{\ast }}{3{k}^{\ast }}\frac{{T}^{3}}{{(\rho {c}_{p})}_{f}})\frac{\partial T}{\partial y}]+\tau [{D}_{B}(\frac{\partial C}{\partial y}\frac{\partial T}{\partial y})+\frac{{D}_{T}}{{T}_{\infty }}{(\frac{\partial T}{\partial y})}^{2}].$$

Introducing the following transformations

$$\eta =\sqrt{\frac{a}{{\nu }_{f}}y},\,u=axf^{\prime} (\eta ),v=-\sqrt{a{\nu }_{f}}\,f(\eta ),\,N=ax\sqrt{\frac{a}{{\nu }_{f}}}w(\eta ),$$

10 $$\theta (\eta )=\frac{T-{T}_{\infty }}{{T}_{w}-{T}_{\infty }},\,\varphi (\eta )=\frac{C-{C}_{\infty }}{{C}_{w}-{C}_{\infty }}.$$

Making use of Eq. (10), equation of continuity satisfies in an identical manner and Eqs. (3)–(7) along with (9) take the following form

11 $$(1+K)f\prime\prime\prime +ff^{\prime\prime} -{f^{\prime} }^{2}-Mf^{\prime} +Kw^{\prime} +\lambda (\theta +Nr\varphi )=0,$$

12 $$(1+\frac{K}{2})w^{\prime\prime} +fw^{\prime} -wf^{\prime} -K(2w+f^{\prime\prime} )=0,$$

13 $$[(1+Rd{(1+(Tr-1)\theta )}^{3})\theta ^{\prime} ]^{\prime} +{\rm{\Pr }}(f\theta ^{\prime} +Nb\theta ^{\prime} \varphi ^{\prime} +Nt{\theta ^{\prime} }^{2})=0,$$

14 $$\varphi ^{\prime\prime} +Scf\varphi ^{\prime} +\frac{Nt}{Nb}\theta ^{\prime\prime} -ASc\varphi {(1+\delta \theta )}^{n}{e}^{(\frac{-E}{1+\delta \theta })}=0,$$

$$f(0)=0,\,f^{\prime} (0)=1,\,w(0)=-mf^{\prime\prime} (0),\,\theta (0)=1-t,\,\theta (0)=1-s,$$

15 $$f^{\prime} (\eta )\to 0,\,{\rm{w}}(\eta )\to 0,\,\theta (\eta )\to 0,\,\varphi (\eta )\to 0\,as\,\eta \to \infty .$$

In these expressions *K*, *M*, *λ*, *Nr*, *Rd*, *Tr*, *Pr*, *Nb*, *Nt*, *Sc*, *A*,*λ*, *E*, *t* and *s* are the micropolar material parameter, magnetic parameter, mixed convection parameter, the Buoyancy ratio parameter, radiation parameter, temperature ratio parameter, Prandtl number, Brownian motion parameter, thermophoresis parameter, Schmidt number, chemical reaction parameter, temperature difference parameter, dimensionless activation energy, thermal and solutal stratification parameters respectively. Mathematically these parameters are expressed in the following manner:

16 $$\{\begin{array}{l}K=\frac{k}{\mu },\,M=\frac{\sigma {{B}_{0}}^{2}}{{\rho }_{f}a},\,\lambda =\frac{{G}_{r}}{{{\rm{Re}}}^{2}},\,Gr=\frac{g\beta (1-{C}_{\infty })({T}_{w}-{T}_{\infty }){x}^{3}}{{{\nu }_{f}}^{2}},\\ {\rm{Re}}=\frac{a{x}^{2}}{{\nu }_{f}},\,Nr=\frac{({\rho }_{p}-{\rho }_{f})({C}_{w}-{C}_{\infty })}{(1-{C}_{\infty })({T}_{w}-{T}_{\infty }){\rho }_{f}\beta },\,Rd=\frac{16{\sigma }^{\ast }{{T}_{\infty }}^{3}}{3{k}^{\ast }{k}_{f}},\,Tr=\frac{{T}_{w}}{{T}_{\infty }},\\ {\rm{\Pr }}=\frac{{\upsilon }_{f}}{\alpha },\,Nb=\frac{\tau {D}_{B}({{\rm{C}}}_{w}-{{\rm{C}}}_{\infty })}{{\nu }_{f}},\,Nt=\frac{\tau {D}_{T}({T}_{w}-{T}_{\infty })}{{T}_{\infty }{\nu }_{f}},\,Sc=\frac{{\nu }_{f}}{{D}_{B}},\\ A=\frac{{{K}_{r}}^{2}}{a},\,\delta =\frac{({T}_{w}-{T}_{\infty })}{{T}_{\infty }},\,E=\frac{{E}_{a}}{{K}_{1}{T}_{\infty }},\,t=\frac{d}{b},\,s=\frac{e}{c}.\end{array}$$

The skin friction *C*_*f*_ local Nusselt number *Nu*_*x*_ and local Sherwood number *Sh*_*x*_ are stated as

17 $${C}_{f}=\frac{{({\tau }_{w})}_{y=0}}{\frac{1}{2}{\rho }_{f}{{U}_{w}}^{2}},N{u}_{x}=\frac{x{q}_{w}}{{k}_{f}({T}_{w}-{T}_{\infty })},S{h}_{x}=\frac{x{j}_{m}}{{D}_{{\boldsymbol{B}}}({{\rm{C}}}_{w}-{{\rm{C}}}_{\infty })}$$

Here ${\tau }_{w}$ is the wall shear stress, *U*_*w*_, *q*_*w*_ and *j*_*m*_ respectively denote surface velocity, the heat flux and mass flux.

18 $${\tau }_{w}=(\mu +k){(\frac{\partial u}{\partial y})}_{y=0}+k{N}_{y=0},\,{j}_{m}=-{D}_{B}{\frac{\partial C}{\partial y}|}_{y=0},{q}_{m}=-{k}_{f}{\frac{\partial T}{\partial y}|}_{y=0}+{{q}_{r}|}_{y=0}$$

After utilizing (9), Eq. (17) takes the following form

19 $$\begin{array}{c}\frac{1}{2}{{\rm{Re}}}^{\frac{1}{2}}{C}_{f}=[1+(1-m)K]f^{\prime\prime} (0),\,{{\rm{Re}}}^{-\frac{1}{2}}S{h}_{x}=-\varphi ^{\prime} (0)\\ {{\rm{Re}}}^{-\frac{1}{2}}N{u}_{x}=-[1+Rd{(1+(Tr-1)\theta (0))}^{3}]\theta ^{\prime} (0),\end{array}\}$$

where ${\rm{Re}}=\frac{a{x}^{2}}{{\nu }_{f}}$ is local Reynolds number based on the stretching velocity *U*_*w*_. It is pertinent to mention that above defined physical parameters have following characteristics:

i. The skin friction coefficient which gives the friction drag between surface and fluid.
ii. The Sherwood number describes the nanoparticle flux rate in the fluid.
iii. The Nusselt number reports the heat transfer rate.

## Method of solution

The numerical method (RK45) is utilized to solve the reduced non-linear differential equations (11–14) with boundary conditions (15). This method is built-in Maple with command dsolve numeric. This method utilizes both fourth and fifth order Runge Kutta method. The error estimate in this method is determined by subtracting these two values and can be used for adaptive step sizing. Some more details about this technique may be found in^39^. The algorithm of the method is followed as:

$${K}_{0}=f({x}_{i},\,{y}_{i})h,$$

$${K}_{1}=f({x}_{i}+\frac{1}{4}h,\,{y}_{i}+\frac{1}{4}{K}_{0})h,$$

$${K}_{2}=f({x}_{i}+\frac{3}{8}h,\,{y}_{i}+\frac{3}{32}{K}_{0}+\frac{9}{32}{K}_{1})h,$$

$${K}_{3}=f({x}_{i}+\frac{12}{13}h,\,{y}_{i}+\frac{1932}{2197}{K}_{0}-\frac{7200}{2197}{K}_{1}+\frac{7296}{2197}{K}_{2})h,$$

$${K}_{4}=f({x}_{i}+h,\,{y}_{i}+\frac{439}{216}{K}_{0}-8{K}_{1}+\frac{3680}{513}{K}_{2}-\frac{845}{4104}{K}_{3})h,$$

20 $${K}_{5}=f({x}_{i}+\frac{1}{2}h,\,{y}_{i}-\frac{8}{27}{K}_{0}+2{K}_{1}-\frac{3544}{2565}{K}_{2}+\frac{1859}{4104}{K}_{3}-\frac{11}{40}{K}_{4})h,$$

$$\,\,{y}_{i+1}={y}_{i}+\frac{25}{216}{K}_{0}+\frac{1408}{2565}{K}_{2}+\frac{2197}{4104}{K}_{3}-\frac{1}{5}{K}_{4},$$

21 $$\,\,{z}_{i+1}={z}_{i}+\frac{16}{135}{K}_{0}+\frac{6656}{12825}{K}_{2}+\frac{28561}{56430}{K}_{3}-\frac{9}{5}{K}_{4}+\frac{2}{55}{K}_{5},$$

where *y* and *z* are the fourth and fifth order Runge Kutta technique. We have chosen ${\eta }_{{\rm{\max }}}=7$ which approaches the asymptotic values given by the conditions (15). The step size can be determined by the following equation in which $\varepsilon$ is the error control tolerance.

22 $${h}_{new}={h}_{old}{(\frac{{\epsilon }{h}_{old}}{|{z}_{i+1}-{y}_{i+1}|})}^{\frac{1}{4}}.$$

## Results and Discussion

This section is devoted to analyze effects of the assorted parameters involved in the flow problem on flow fields, wall shear stress, heat, and mass transfer. The results are presented in the form of graphs and tables.

**Figure 2:**
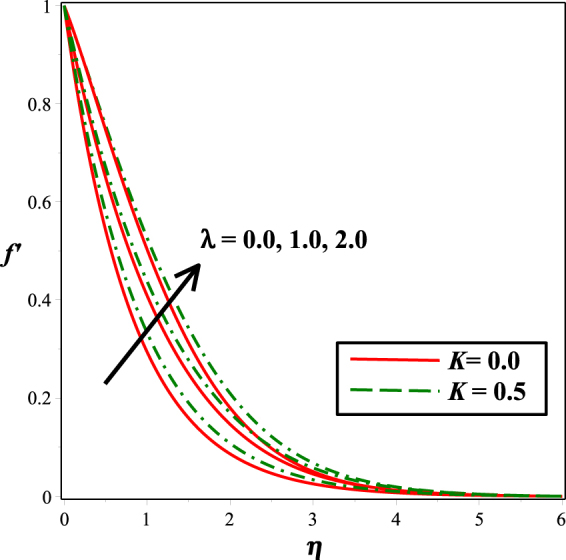
Impact of *λ* on *f* ′(*η*).

**Figure 3:**
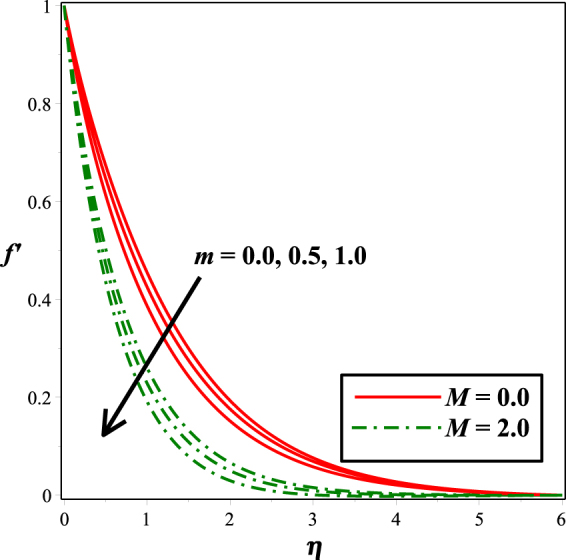
Impact of *m* on *f* ′(*η*).

**Figure 4:**
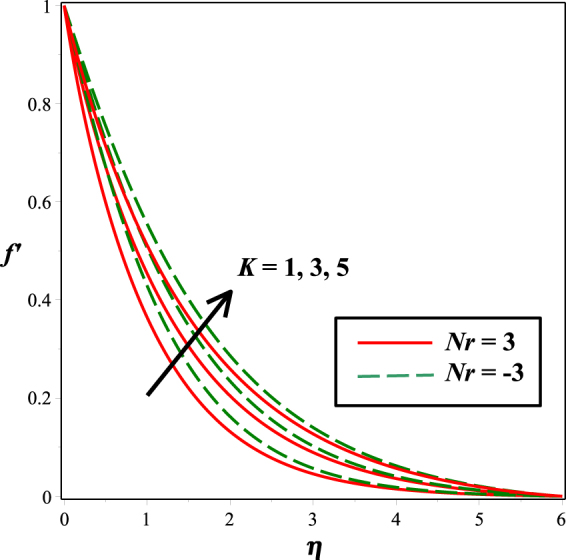
Impact of *K*on *f* ′(*η*).

The effect of mixed convection parameter *λ* on velocity field is captured in Fig. 2. Since an increase in *λ* improves the bouncy forces and therefore increases the velocity. Also, it is noted that presence of micro-rotation gives higher values than that of the Newtonian fluid (*K* = 0). Figure 3 is plotted for the impact of micro-gyration parameter *m* on fluid flow. It is noticed that inflating *m* from 0 to 1, the fluid motion enhances. This effect is observed under the action and in the absence of magnetic field, and smaller values are seen for the velocity in the presence of magnetic field. The influence of micropolar material parameter *K* on velocity field is sketched in Fig. 4. From the figure, it can be deduced that the fluid motion is an increasing function of escalating values of *K*.

**Figure 5:**
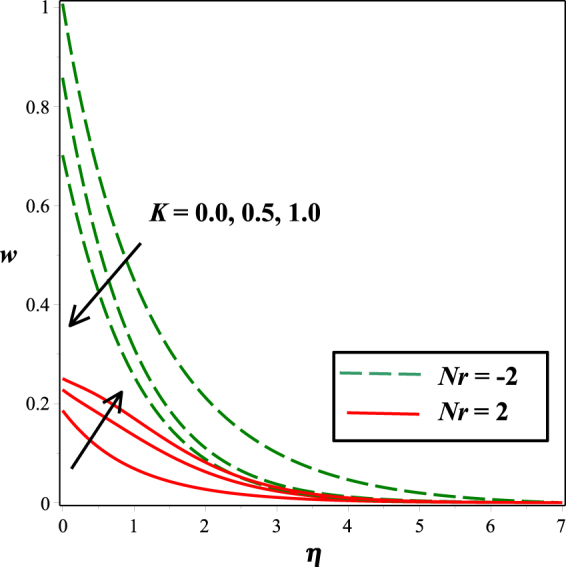
Impact of *K* on *w*(*η*).

**Figure 6:**
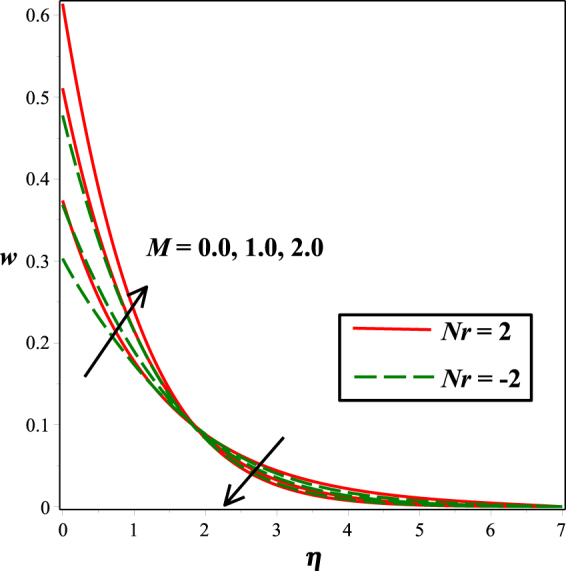
Impact of *M* on *w*(*η*)*w*(*η*).

**Figure 7:**
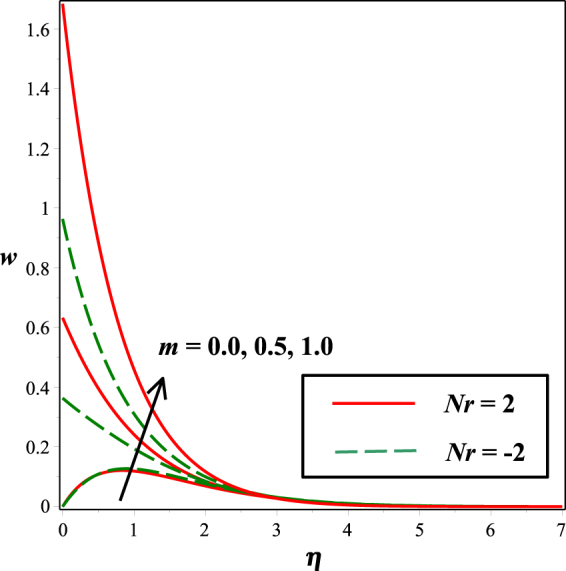
Impact of *m* on *w*(*η*).

**Figure 8:**
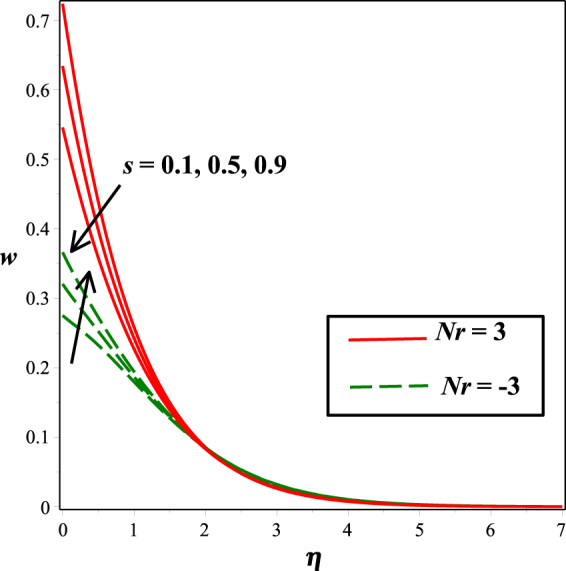
Impact of *s* on *w*(*η*).

**Figure 9:**
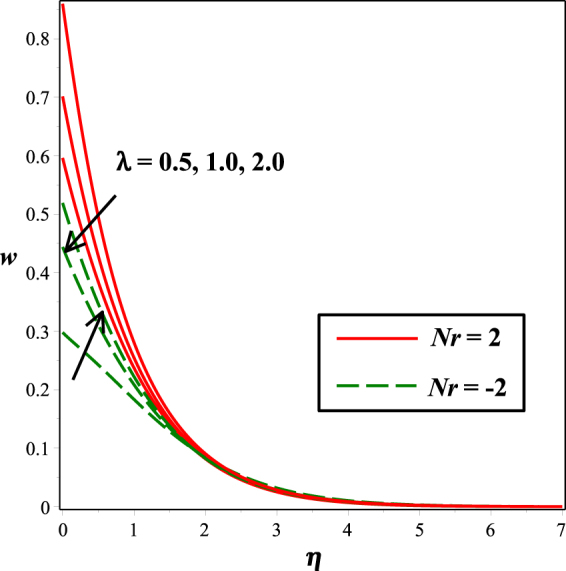
Impact of *λ* on *w*(*η*).

The effects of numerous parameters on the micro-rotation component are captured in Figs 5, 6, 7, 8, 9. From Fig. 5, the influence of material parameter *K* on micro-rotation distribution is observed. Increasing the values of *K*, the micro-rotation component develops for the aiding buoyancy forces (*Nr* > 0) and diminishes for opposing buoyancy forces (*Nr* < 0). The variation in micro-rotation distribution due to magnetic field *M* effect is displayed in Fig. 6. It is seen that micro-rotation field increase near the surface and decreases away from the surface by enhancing the effect of *M*. The impact of micro-gyration parameter *m* on angular velocity component is presented in Fig. 7. From boundary conditions (14), *m* = 0 indicates a strong concentration of particles, i.e., the case in which micro-elements closer to the surface are unable to rotate. *m* = 0.5 represents weak concentration in which symmetric part of the stress tensor vanishes. The case *m* = 1 interprets turbulent boundary layer flows and in this case the angular velocity component is dominant. The behavior of micro-rotation component for raising values of solutal stratification parameter *s* can be seen in Fig. 8. It is observed that *s* decreases for aiding buoyancy forces and enhances for opposing buoyancy forces. Similar behavior is observed from Fig. 9 with growing values of mixed convection parameter *λ*.

**Figure 10:**
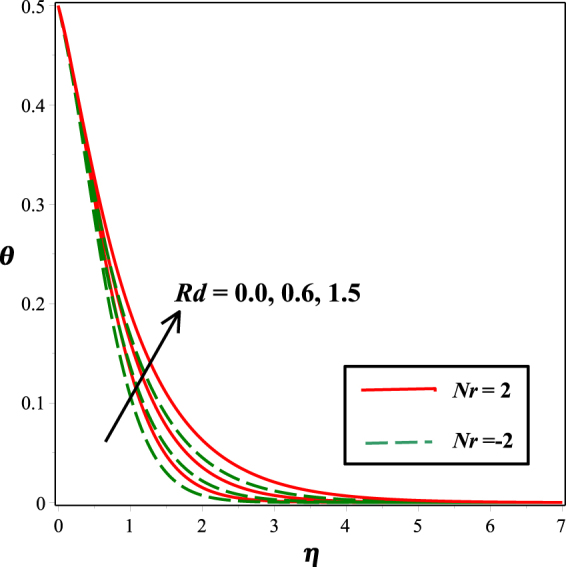
Impact of *Rd* on *θ*(*η*)*θ*(*η*).

**Figure 11:**
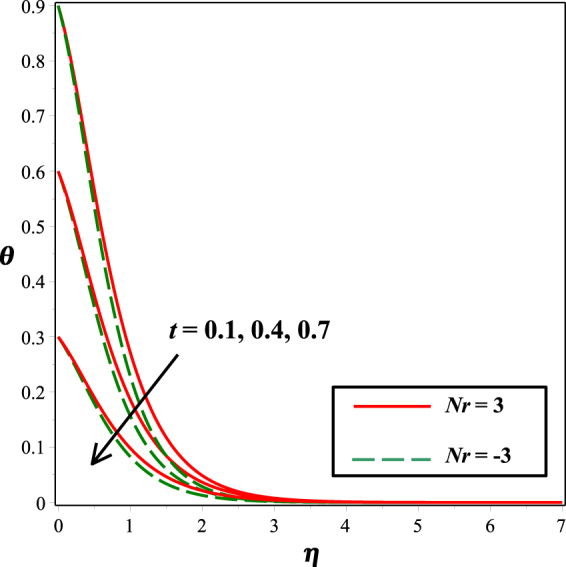
Impact of *t* on *θ*(*η*).

The incremented behavior of temperature profile due to raising radiation parameter *Rd* is considered in Fig. 10. As radiation parameter *Rd* indicates the relative contribution of conduction heat transfer to radiation heat transfer. Increasing estimations of *Rd* increases heat transfer rate and related thermal boundary layer, so, more heat is transferred to the fluid which results in increased temperature. From Fig. 11, it is noticed that temperature field decay for mounting values of thermal stratification parameter *t*. As by considering temperature variation, stratification between the surface and the ambient fluid decreases, therefore, temperature reduces.

**Figure 12:**
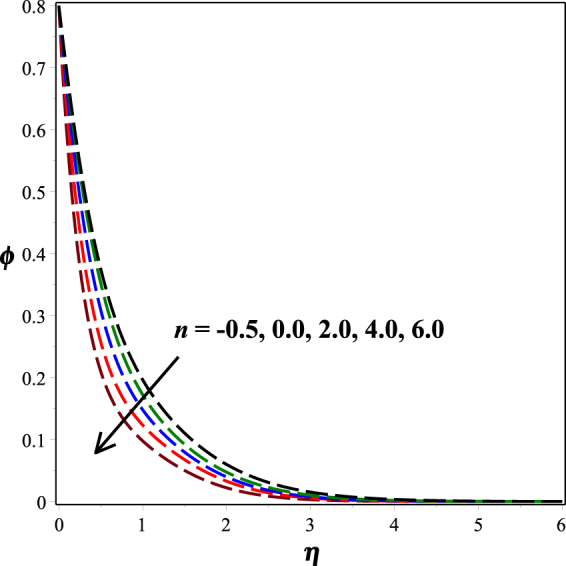
Impact of *n* on *ϕ*(*η*).

**Figure 13:**
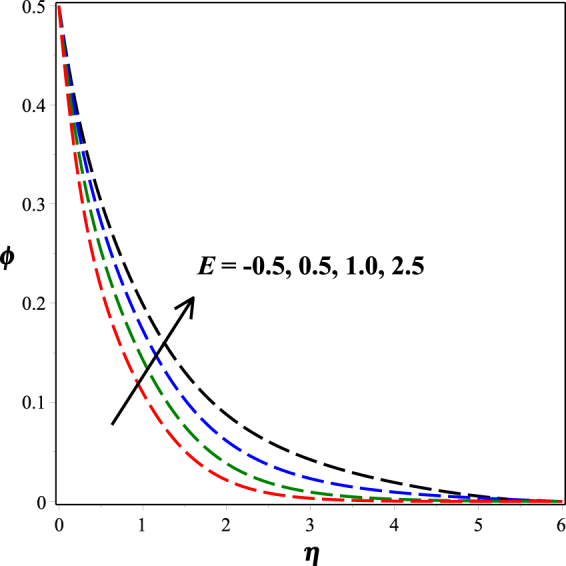
Impact of *E* on *ϕ*(*η*).

**Figure 14:**
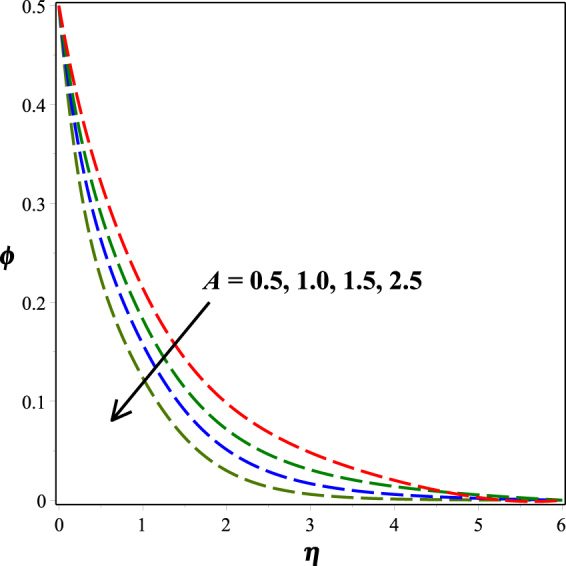
Impact of *A* on *ϕ*(*η*).

**Figure 15:**
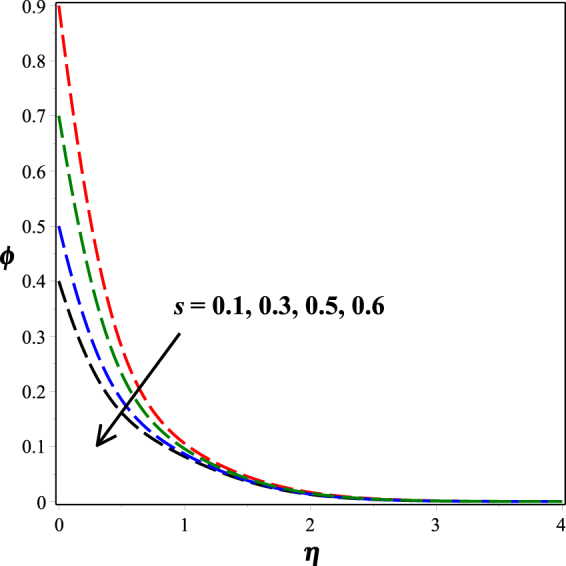
Impact of *s* on *ϕ*(*η*).

Figure 12 portrays the impact of fitted rate constant *n* on nanoparticle concentration distribution. It is seen that as *n* ascents, concentration profile descents. The influence of dimensionless activation energy *E* on nanoparticle concentration is graphed in Fig. 13. Increase in activation energy boosts the constructive chemical reaction and in turn raise the concentration of the nanoparticles is observed. Figures 14 and 15 present the diminishing behavior of concentration distribution along increasing chemical reaction rate constant *A* and solutal stratification parameter *s*.

**Figure 16:**
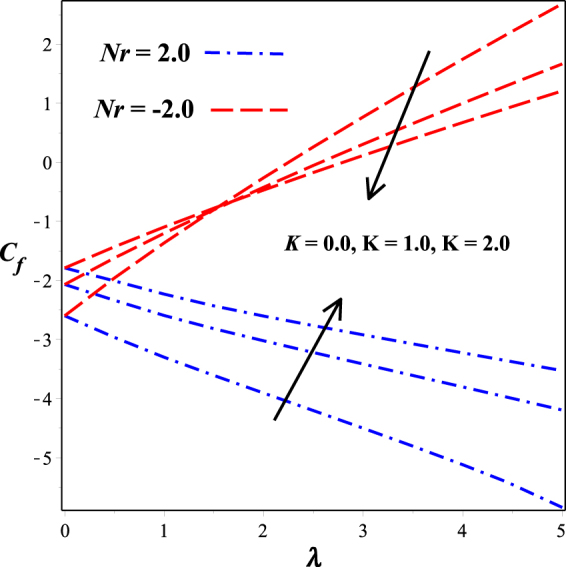
Impact of K on *C*_*f*_ against *λ*.

**Figure 17:**
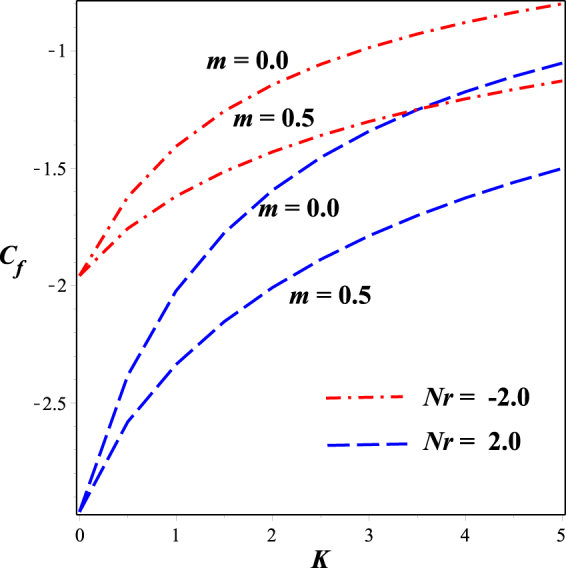
Impact of m on *C*_*f*_ against *K*.

Figures 16 and 17 depict the behavior dimensionless surface shear stress. Figure 16 is sketched against mixed convection parameter *λ* for varying micropolar material parameter *K*. It is seen that Skin friction coefficient declines in the opposing flow (*Nr* > 0) and escalates for aiding flow (*Nr* > 0) From Fig. 17, we observed that the surface shear stress decreases while moving from strong concentration (m = 0.0) towards weak concentration (m = 0.5). This effect is plotted against the material parameter *K* both in the presence of aiding and opposing flow.

In addition to graphical results, the numerical values of dimensionless heat and mass transfer rate for varying different parameters are included in Tables 1 and 2. In Table 1 the numerical data of local Nusselt number is presented for different values of $K,\,Rd,\,Tr,\,{\rm{\Pr }},\,n,\,E\,{\rm{and}}\,t\,\,$fixing the other parameters. The effects of these parameters are observed both in strong (m = 0) and weak concentration (*m* = 0.5). From Table 1, we can see that heat transfer rate enhances for the increasing values of fitted rate constant *n* and thermal stratification parameter *t* while it shows decreasing behavior for all other varying parameters for both strong and weak concentration. Table 2 includes the value of Sherwood number both in strong and weak concentration of nanoparticles. The changes in mass transfer rate for varying *K*, *Sc*, *A*, *δ*, *E*, *n*, *s and Nb* are reported. It is observed that the concentration transfer gives descending values for activation energy *E*, and thermal stratification *s*, whereas it shows incremented behavior for all other parameters.

**Table 1:** Numerical values of the Nusselt number for different values of *K*, *Rd*, *Tr*, *t*, *n*, *E* and Pr.

*K*	*Rd*	*Tr*	*t*	*n*	*E*	Pr	${\bf{R}}{{\bf{e}}}^{{\boldsymbol{-}}\frac{{\bf{1}}}{{\bf{2}}}}{\boldsymbol{N}}{{\boldsymbol{u}}}_{{\boldsymbol{x}}}$	
							*m* = 0.0	*m* = 0.5
0.0	0.5	0.7	0.5	1.0	1.0	2.0	0.25853	0.25853
1.0							0.31100	0.28891
2.0							0.33718	0.31056
1.0	0.4	0.7	0.5	1.0	1.0	2.0	0.29685	0.27590
	0.8						0.35016	0.32523
	1.5						0.42917	0.39997
1.0	0.5	0.5	0.5	1.0	1.0	2.0	0.27741	0.25813
		1.0					0.311001	0.28891
		2.0					0.40634	0.37622
1.0	0.5	0.7	0.2	1.0	1.0	2.0	0.311001	0.28891
			0.5				0.30667	0.28609
			0.8				0.30354	0.28389
1.0	0.5	0.7	0.5	0.5	1.0	2.0	0.42718	0.40043
				1.0			0.29033	0.26986
				2.0			0.12583	0.11586
1.0	0.5	0.7	0.5	1.0	0.0	2.0	0.28457	0.26566
					1.0		0.29418	0.27249
					2.0		0.29746	0.22745
1.0	0.5	0.7	0.5	1.0	1.0	2.0	0.22634	0.21148
						4.0	0.32208	0.30132
						6.0	0.33727	0.32024

**Table 2:** Numerical Values of Sherwood number for different values of the emerging parameters *K*, *Sc*, *δ*, *n*, *s*, *A*, *E* and *Nb*.

*K*	*Sc*	*δ*	*n*	*s*	*A*	*E*	*Nb*	${\bf{R}}{{\bf{e}}}^{{\boldsymbol{-}}\frac{{\bf{1}}}{{\bf{2}}}}{\boldsymbol{S}}{{\boldsymbol{h}}}_{{\boldsymbol{x}}}$	
								*m* = 0.0	*m* = 0.5
0.0	1.0	0.5	1.0	0.2	0.4	1.0	0.5	0.45565	0.45565
1.0								0.48025	0.46947
2.0								0.49116	0.48013
1.0	2.0	0.5	1.0	0.2	0.4	1.0	0.5	0.80638	0.78511
	4.0							1.26455	1.24112
	6.0							1.60863	1.58495
1.0	1.0	0.4	1.0	0.2	0.4	1.0	0.5	0.47130	0.45973
		0.8						0.50686	0.49820
		1.2						0.54168	0.53536
1.0	1.0	0.5	−0.5	0.2	0.4	1.0	0.5	0.44206	0.42990
			1.0					0.48025	0.46947
			2.0					0.50840	0.49997
1.0	0.5	0.5	1.0	0.3	0.4	1.0	0.5	0.41257	0.40453
				0.5				0.27215	0.26896
				0.7				0.12552	0.12635
1.0	0.5	0.5	1.0	0.2	0.5	1.0	0.5	0.52108	0.51195
					1.0			0.68425	0.67934
					1.5			0.81176	0.80861
1.0	0.5	0.5	1.0	0.2	0.5	2.0	0.5	0.36175	0.34494
						4.0		0.25122	0.22317
						6.0		0.22293	0.18868
1.0	0.5	0.5	1.0	0.2	0.5	1.0	0.3	0.42484	0.42510
							0.6	0.49533	0.48193
							0.9	0.52169	0.50430

A comparison table for validation of our present computations is also presented in Table 3, a good agreement with previous results in^38^ is achieved.

**Table 3:** Comparison table for the Nusselt number for different values of Pr, *Nt*, *E*, *A*, *n* and *λ* setting *K* = *Rd* = *Tr* = s = *t* = 0.

Pr	*Nt*	*E*	*A*	*n*	*λ*	−θ′(0)	
						Mustafa *et al*.^38^	Present
2.0	0.5	1.0	1.0	0.5	0.5	0.706605	0.706615
4.0						0.935952	0.935943
7.0						1.132787	1.132296
10						1.257476	1.257213
5.0	0.1	1.0	1.0	0.5	0.5	1.426267	1.426028
	0.5					1.013939	1.013819
	0.7					0.846943	0.846943
	1.0					0.649940	0.649911
5.0	0.5	0.0	1.0	0.5	0.5	0.941201	0.941201
		1.0				1.013939	1.013912
		2.0				1.064551	1.064493
		4.0				1.114549	1.114329
5.0	0.5	1.0	0.0	0.5	0.5	1.145304	1.145299
			1.0			1.013939	1.013912
			2.0			0.926282	0.926264
			5.0			0.798671	0.798653
5.0	0.5	1.0	2.0	−1.0	0.5	1.030805	1.03800
				−0.5		0.999470	0.999468
				0.0		0.964286	0.964286
				1.0		0.886830	0.886829
5.0	0.5	1.0	2.0	0.5	0.0	1.032281	1.032281
					0.5	1.056704	1.056701
					3.0	1.154539	1.154527
					5.0	1.215937	1.215932

## Concluding remarks

The effects of heat transfer, Rosseland thermal radiation in the flow of micropolar nanofluid with chemical reaction and activation energy are considered here. The condition of thermal and solutal stratification is also taken into account. The problem is then solved numerically using Runge-Kutta fourth and fifth order technique which is built-in Maple differential solver command. The following remarks are deduced from our study:

- Velocity is an increasing function of micropolar parameter *K*.
- Velocity field diminishes for enhancing values of buoyancy ratio parameter *N*_*r*_.
- Micro-rotation distribution decreases for aiding buoyancy forces and increases for opposing flow with increase in values of solutal stratification parameter *s*.
- Temperature profile shows increasing behavior and diminishes for raising the values of radiation parameter *Rd* and thermal stratification *t*.
- The concentration profile enhances for the improving values of activation parameter *E* It descends for rising values of solutal stratification parameter *s*.
- The skin friction coefficient decreases for opposing flow and increases for aiding flow by changing the values of the micropolar material parameter *K*.
- Heat transfer rate improves for escalating activation energy parameter *E* and it shows decreasing behavior for escalating thermal stratification *E*.
- Concentration profile ascents for escalating chemical reaction parameter *A* while it diminishes for enhancing solutal stratification parameter *s*.
